# NetworkAnalyst - integrative approaches for protein–protein interaction network analysis and visual exploration

**DOI:** 10.1093/nar/gku443

**Published:** 2014-05-26

**Authors:** Jianguo Xia, Maia J. Benner, Robert E. W. Hancock

**Affiliations:** 1 Department of Microbiology and Immunology, University of British Columbia, Vancouver, Canada; 2 Centre for Microbial Diseases and Immunity Research, University of British Columbia, Vancouver, Canada; 3 Wellcome Trust Sanger Institute, Hinxton, United Kingdom

## Abstract

Biological network analysis is a powerful approach to gain systems-level understanding of patterns of gene expression in different cell types, disease states and other biological/experimental conditions. Three consecutive steps are required - identification of genes or proteins of interest, network construction and network analysis and visualization. To date, researchers have to learn to use a combination of several tools to accomplish this task. In addition, interactive visualization of large networks has been primarily restricted to locally installed programs. To address these challenges, we have developed NetworkAnalyst, taking advantage of state-of-the-art web technologies, to enable high performance network analysis with rich user experience. NetworkAnalyst integrates all three steps and presents the results via a powerful online network visualization framework. Users can upload gene or protein lists, single or multiple gene expression datasets to perform comprehensive gene annotation and differential expression analysis. Significant genes are mapped to our manually curated protein-protein interaction database to construct relevant networks. The results are presented through standard web browsers for network analysis and interactive exploration. NetworkAnalyst supports common functions for network topology and module analyses. Users can easily search, zoom and highlight nodes or modules, as well as perform functional enrichment analysis on these selections. The networks can be customized with different layouts, colors or node sizes, and exported as PNG, PDF or GraphML files. Comprehensive FAQs, tutorials and context-based tips and instructions are provided. NetworkAnalyst currently supports protein-protein interaction network analysis for human and mouse and is freely available at http://www.networkanalyst.ca.

## INTRODUCTION

High-throughput omics technologies have enabled global measurement of biological molecules (DNA, RNA, proteins, metabolites, etc.) under various experimental conditions and disease states. The strategies for obtaining systems-level understanding, based on these datasets, have become an active research area in bioinformatics and computational biology over the past decade. Many powerful approaches have been proposed and implemented to provide higher-level summaries in terms of gene ontologies (GO), pathways, gene sets or network modules (1–4). In particular, network-based approaches show promise of providing the most unbiased analysis. Among different molecular networks, protein–protein interaction (PPI) networks have emerged as an important resource for understanding data from gene expression or proteomics experiments. Protein interactions play fundamental roles in structuring and mediating essentially all biological processes. PPI can be derived from small- to large-scale experiments or computational predictions (5,6). PPI networks are often presented as undirected graphs with nodes as proteins and edges indicating interactions between two connecting proteins.

Three consecutive steps are typically required to perform PPI network analysis. The first step is to identify genes or proteins of interest. Common choices include differentially expressed genes, mutated genes, genes with copy number variations, genes with single nucleotide polymorphisms, genes targeted by microRNAs, etc. In the second step, these inputs (also known as ‘seed proteins’) are used to search and retrieve binary interactions from a curated PPI database. A network can be assembled based on the set of interactions, which is usually composed of co-regulated nodes and nodes that they are known to interact with (first-order interactors). The third step is network analysis. Two complementary approaches are often performed - the topology analysis that considers the whole network structure to search for important nodes (hubs) which are useful as biomarkers or therapeutic targets, and module analysis that breaks the complex network into small densely connected units (modules) and aims to identify the ones showing more activities (or active ‘hotspots’). However, despite the increasing researches in the past few years, there is no consensus or standard solutions regarding how to define and identify hubs or modules in biological networks. Therefore, results from network analysis should be visually inspected and further validated by other well-established approaches such as GO or pathway enrichment analysis.

A variety of tools have been developed to help bench researchers analyze and visualize their data generated from different omics experiments within the context of biological networks (7–12). Among them, Cytoscape and its plugins have provided a powerful toolkit that can perform a wide range of functions for network analysis and visualization (13). However, effective use of Cytoscape requires a good understanding of the tool and plugins available as well as skills in organizing and interpreting the output. In addition, almost all these tools have been implemented as Java-based graphical user interface (GUI) programs, and run as standalone desktop applications or as embedded Applets. The former requires users to install the programs locally and to maintain the compatibility between different versions and plugins, while the latter approach is associated with security concerns and the lack of universal support in browsers. In addition, these specialized programs usually do not have good support for analysis of gene expression data, which is among the most common input types. As a result, researchers require a combination of several tools to perform network analysis. There is a clear need for facile, point-and-click web-based tools that allow bench researchers to seamlessly move from their gene expression data to network analysis and visualization, without having to use and install multiple different tools.

The advance of web-based technologies such as HTML5 and JavaScript, together with ever-increasing computing power available through modern web browsers, has made it possible to develop high-performance web-based visualization tools (14–17). In this paper, we introduce NetworkAnalyst, a web-based user-friendly tool that integrates all essential steps of network analysis, and present the results through a powerful online visualization system. The key features of NetworkAnalyst includes:

- support of gene/protein list and single/multiple gene expression data;
- flexible differential expression analysis for a variety of experimental designs;
- multiple procedures to control network size during network construction;
- interactive network visualization with facile searching, zooming and highlighting;
- support of topology, module and shortest-path analysis;
- functional enrichment analysis (GO, KEGG and Reactome) on current selections;
- customization with different layouts, edge shapes and node sizes/colors/visibility;
- network editing including node deletion and module extraction;
- downloading of output as network files (edge list, graphML), images (PNG, PDF) and topology/functional analysis results.

NetworkAnalyst is designed to perform efficient PPI network analysis for data generated from gene expression experiments from human and mouse studies. The current implementation allows analysis and rapid visualization of the resulting PPI networks from small to large size (100–1000s nodes). NetworkAnalyst is freely available at www.networkanalyst.ca.

## PROGRAM DESCRIPTION AND METHODS

**Figure 1.**
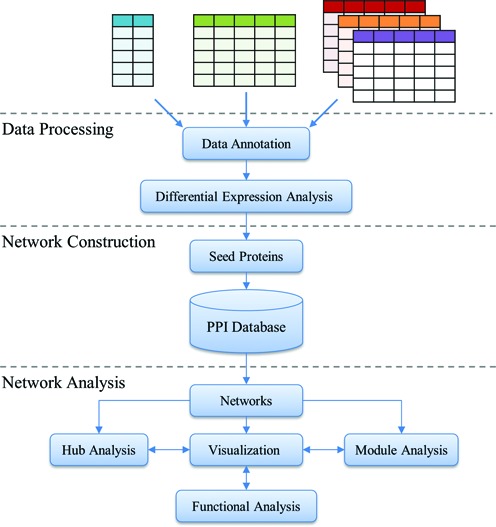
NetworkAnalyst workflow. NetworkAnalyst accepts a gene/protein list, single or multiple gene expression datasets. Three consecutive steps are performed - data processing, network construction and network analysis. The resulting network can be visually explored through an online interactive visualization system.

NetworkAnalyst was developed around the three major steps in network analysis - a data processing step to identify significant genes; a network construction step for mapping, building and refining networks; and a network analysis and visualization step. Figure 1 shows the flowchart of NetworkAnalyst. For each step, multiple options are provided with detailed instructions and help information. Upon completion of each step, the system also provides dynamic feedback messages to give further user guidance. For the first-time users, it is suggested to start with the inbuilt tutorials to become familiar with the basic features and main steps. Multiple example datasets are also provided to allow users to explore the various features. We have also prepared a comprehensive list of frequently asked questions (FAQs) to provide tips as well as technical details about the algorithms used in the analysis.

### Data processing

#### Data formats and uploading

Clicking the ‘Start’ menu on the home page allows users to enter the data processing page. Users then choose from three types of input - gene/protein list, a single gene expression dataset or multiple gene expression datasets. NetworkAnalyst accepts a list of genes/proteins IDs with optional fold change values separated by a tab space. The gene expression data is uploaded as a tab delimited text file (.txt) or in its compressed form (.zip) with genes/probes in rows and samples in columns. The row containing class labels must begin with #CLASS. Multiple experimental factors (meta-data) can be included in separate rows. For instance, if users want to analyze their data with regard to both disease conditions and time points, they should label these two corresponding rows with #CLASS:disease and #CLASS:time. The FAQs page contains detailed descriptions, as well as example datasets for illustration purposes.

#### Data processing and annotation

After a dataset is successfully uploaded, users annotate the data by converting different feature IDs to a common ID. When multiple probes are mapped to the same gene, the results will be presented as an average of combined probes. NetworkAnalyst currently supports five types of common gene IDs (Entrez, RefSeq, GenBank, Ensembl gene and transcript), official gene names and 45 probe-set IDs corresponding to 45 microarray platforms for human and mouse.

#### Data normalization and analysis

Data can be further normalized to log_2_ scale (microarray) or log_2_ counts per million (RNA-seq). NetworkAnalyst can deal with very complex study designs for microarray and RNAseq experiments based on the well-established linear models (18). We have recently created a user-friendly interface to allow users to set various parameters to take advantage of this powerful and flexible approach (15). The interface currently supports two-group or multiple-group comparisons involving paired or block design, time-series, common-control and nested-comparisons. When there are multiple meta-data, users need to first indicate the meta-data of interest, and then set the comparisons of interest. NetworkAnalyst allows users to select significant genes using a combination of *P* value and log fold change cutoffs.

### Network construction

NetworkAnalyst uses a comprehensive high-quality PPI database downloaded from the InnateDB (19), which participates in the International Molecular Exchange (IMEx) consortium (20). The database was created by manually curating protein interaction data from published literature as well as by integrating experimental data from several PPI databases including IntAct (21), MINT (22), DIP (23), BIND (24) and BioGRID (25). The database contains 14 755 proteins and 145 955 experimentally confirmed interactions for human, and 5657 proteins and 14 491 interactions for mouse.

For each individual (seed) protein, a search algorithm is performed to identify proteins that directly interact with the seed proteins (first-order interactors). The results are used to build the default networks. This approach will typically return one large subnetwork (‘continent’) with several smaller ones (‘islands’). Most subsequent analyses are performed on the continent. We recommend users to control the number of nodes to be in the range of 200 - 2000 for practical (visual, biological and computational) reasons since larger networks will lead to a ‘hairball’ effect, which rarely produces any informative outcome, while smaller networks will not enable systems-level understanding.

Several functions have been implemented to allow users to further adjust the network size. For instance, if the default network constructed with first-order interactors is small (i.e. <100 nodes), users can search for higher-order interactions by treating all nodes in this network as new seed proteins. Users can also trim a large network to be composed only of nodes that connect the original seed proteins. When there are too many seed proteins (i.e. >1000) and the default network will be too large to be visualized, users can choose to focus only on networks within these seed proteins (zero-order interactors). In addition, NetworkAnalyst allows users to adjust the cutoffs for *P* values or fold changes to control the number of seed proteins, as well as to manually exclude certain proteins that are known to be involved in many non-specific interactions, to enable fine control of network size.

### Network analysis

**Figure 2.**
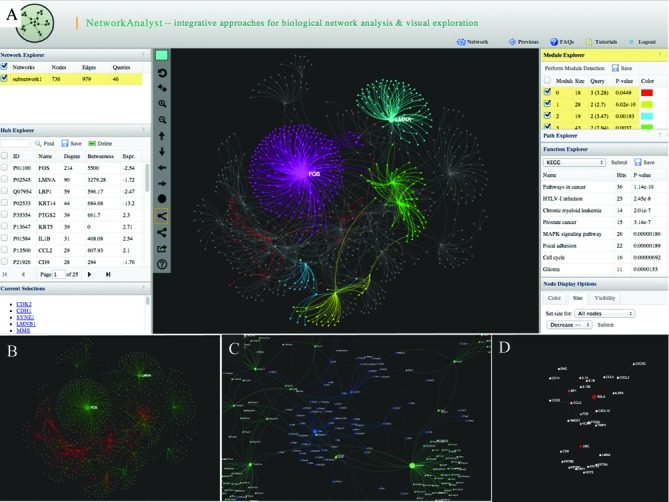
Some screenshots of NetworkAnalyst. (**A**) The screenshot shows a typical view of network analysis and visualization. The center panel shows the network with multiple modules highlighted in different colors. Several surrounding panels including *Network Explorer*, *Hub Explorer*, *Module Explorer*, *Path Explorer* and *Function Explorer* allow users to perform various analyses; (**B**) an expression view shows the overall expression pattern, with red for up-regulation and green for down-regulation; (**C**) a zoom-in view shows part of a dense network. Nodes are labeled automatically; (**D**) a module extracted from a large network using the top 30 nodes with highest degrees.

Substantial efforts have been devoted to implement a facile and intuitive framework for network analysis and visualization. Figure 2 shows a screenshot of the interface. The network is displayed at the center, with five major surrounding panels—*Network Explorer*, *Hub Explorer*, *Module Explorer*, *Path Explorer* and *Function Explorer*. The network analyses are performed primarily using the functions within these panels.

*Network Explorer* shows all networks constructed from the seed proteins. The numbers of nodes, edges and seed proteins are summarized for each network. Clicking a row allows visualization of the corresponding network. The *Hub Explorer* contains detailed information of nodes within the current network. To help identification of highly interconnected hub nodes, NetworkAnalyst provides two widely used topological measures—degree and betweenness centrality (26). The degree of a node is the number of connections it has to other nodes. The betweenness centrality measures number of shortest paths going through the node. Nodes with higher degree or betweenness values are potentially important hubs in cellular signal trafficking. The *Expr.* column shows the log fold change values of the corresponding node if it is a seed protein. For meta-analysis, the value is the number of times it has been identified as significant in different datasets. A ‘–’ symbol indicates the node is not a seed protein. Clicking on a column header results in sorting of the table accordingly. Clicking on a node row leads to zooming in on the selected node within the network. Multiple nodes can be selected using the checkbox. These features allow users to easily visualize all seed proteins or the top *n* nodes with highest degrees or betweenness values, by first sorting and then selecting these nodes.

The *Module Explorer* allows users to decompose the current network into smaller densely connected modules, which are considered as relatively independent components such as pathways or protein complexes. Nodes within a module are likely to work collectively to perform a biological function. NetworkAnalyst uses the well-established Walktrap algorithm based on random walks (27). The general idea is that if we perform random walks on the network, the walks are more likely to stay within the same module because there are only a few edges that lead outside a given module. Initially, every node belongs to a separate module. The Walktrap program then runs multiple short random walks and uses the results of these random walks to merge separate modules in a bottom-up manner. NetworkAnalyst uses a weighted network for module detection with edge weights calculated as the square of mean absolute log fold changes of the two adjacent nodes (28). The resulting table shows the identified modules ranked by the numbers of seed proteins they contain. The *P* value indicates the significance of each module by testing the difference between the number of edges within a module and the edges connecting the nodes of a module with the rest of the network based on Wilcoxon rank-sum test. The number of seed proteins (query) in each module is also provided together with their average log fold changes. Clicking a module row leads to highlighting the corresponding module within the network. Users can set different colors for different modules as described below in the Network visualization section.

*Path Explorer* allows users to detect the shortest paths between any two given nodes. Clicking any path in the result list enables one to highlight the path within the current network. *Function Explorer* allows users to perform functional enrichment analysis for currently highlighted nodes using different databases such as the GO, KEGG or Reactome pathway databases. A hypergeometric test is used to compute the enrichment *P* values. The total matched proteins are also shown. These nodes can be highlighted within the network by double clicking the corresponding row.

### Network visualization

#### Network navigation using a mouse

We recommend using a mouse with a scroll wheel. The mouse events are described below:

- Hovering the mouse over any node will show only that node and its direct neighbors. This is designed to reduce the hairball effect while navigating a densely connected network. Please note the feature is disabled by default when a network contains less than 1000 nodes. Users can activate the ‘Mouse-over effect’ on *Node display options*.
- With the mouse over a node, one can use the scroll wheel to zoom in and out the network with the node in the center. Depending on the zoom level, node labels will automatically show up or disappear.
- Clicking a node to shows its detailed information on the *Current Selections* panel on the bottom-left corner of the window, with hyperlinks to Uniprot, NCBI and KEGG.
- Double clicking a node highlights it in the currently selected color and also increases its size. Depending on the selection mode (described below), the neighborhood may also be highlighted.
- The mouse can be used to directly drag the network to different positions.

Beside functions for reset, zoom and move the network, the top-left toolbar also contains several important functions. The top colored square (color picker) allows users to set the highlight color for next selection. Users can choose among three highlighting modes - single-node mode, node-edge mode and module mode. The option will affect the highlighting range when double clicking a node. Finally, the currently highlighted module can be extracted from the parent network by clicking the ‘extract module’ icon on the toolbar.

#### Node display options

The panel on the bottom-right contains functions to adjust the colors, sizes and visibility for different node groups. For instance, when seed proteins from multiple datasets are projected to the network, users can choose to highlight those nodes from one particular data. For each data, users can further select up-regulated or down-regulated nodes. Node sizes can also be adjusted to further highlight the current selections. Finally, users can choose to enable/disable mouse-over effect, or to hide those nodes not in highlight in order to reduce cluttering due to the overlaps in a dense network.

#### Network options

The ‘Network’ menu on top of the window contains common functions for network manipulation. The *Default View* controls the default color scheme of the network, with *Topo Highlight* coloring based on node degrees and *Expression Highlight* based on node expression levels. The *Layout* controls the overall network layout. NetworkAnalyst supports several force-directed layout algorithms including *ForceAtlas*, *YifanHu* and *Fruchterman-Rengold*, based on the widely acclaimed Gephi implementation (29). The default layout is generated using *ForceAtlas* followed by *YifanHu* layout, which usually produces satisfactory results. Users can also choose to switch between curve (default) and straight line for edge shape. The *Export* submenu allows users to save the current network in GraphML, PNG or PDF format.

#### Node deletion and module extraction

To remove nodes, users need to first select them from the *Hub Explorer* table, and then click the ‘Delete’ button from the toolbar above. To extract a module, users need to first select (highlight) it in the current network, and then click the Extract icon on the toolbar on the left of the Network panel. Network editing is a computationally expensive task as it involves not only the node itself, but also those nodes that it connects with. Deleting an important hub node may also break the network into several pieces.

### Implementation and session management

NetworkAnalyst's web interface was developed using the latest Java Server Faces 2.0 technology. The network visualization was developed based on the *sigma.js* JavaScript library (http://sigmajs.org/). The backend statistical computation was implemented using the R programming language (http://www.r-project.org/). The layout algorithms are based on *Gephi toolkit* (https://gephi.org/toolkit/). The PPI database is stored in an embedded *Neo4j* graph database (http://www.neo4j.org/) for fast search. Each time a user starts a session, a temporary account is created together with a temporary folder to store all user uploaded datasets and analytical results. Users are expected to download all their processed datasets, images and result tables upon completion of a session. NetworkAnalyst allows users to download a file containing the core information of the current session. Users can then upload the saved session file to skip the most tedious and time-consuming data preparation process, and directly jump to network construction and visualization.

NetworkAnalyst is designed to facilitate exploratory data analysis and real-time interaction with the users and is especially designed for biologists with modest computational skills. Results are usually returned in a few seconds to less than a minute. The performance of interactive network visualization is dependent on the users’ browser. NetworkAnalyst has been tested with major modern browsers with HTML5 support, such as Google Chrome (5+), Mozilla Firefox (3+) and Microsoft Internet Explorer (9+). For best user experience, we recommend using a computer with at least 2G RAM and 1280 × 800 screen resolution.

## LIMITATIONS AND FUTURE DIRECTIONS

Network analysis is a very complex task and users need to be wary of several important pitfalls and limitations. Firstly, the majority of PPI data are derived from high-throughput experiments using techniques such as yeast two-hybrid screens or protein pull-down followed by mass spectrometry. These data are very noisy in nature and can contain many false positives, although the current drive to use protein interaction data standards through the IMEx consortium is reducing this issue (20). The results from the network analysis should be used for hypothesis generation and for the design of further validation experiments. Secondly, a major goal of network analysis is to identify and extract interactions from static networks that appear to be active under the new experimental conditions. Increasing evidence has shown that biological networks are dynamic and undergo re-wiring during different conditions and are impacted by the specific cell type under consideration. Therefore, this approach is unable to identify new interactions that are condition-specific (30).

Although NetworkAnalyst has been developed to give high-performance network visualization, we advise users to limit this to less than 5000 nodes. There are two considerations. Firstly, direct visualization of a very large network is ineffective (‘hairball’ effect) and computationally expensive. Secondly, given that there are an average of 10 interaction partners for each protein, the setting will allow ∼500 significant genes or seed proteins to be processed using the default first-order interactions. From here, users can expand the network by including higher-order interactions for very small network; or shrink the network to zero-order interactions when there are too many seed proteins.

NetworkAnalyst has been developed as a web-based alternative to Cytoscape, with universal access and better support for gene expression data analysis. However, given the long history and the large number of plugins developed for Cytoscape, the current implementations of NetworkAnalyst focus on the most popular features of Cytoscape. According to (31), the top two popular Cytoscape plugins are BiNGO (32) and MCODE (33). We have provided the equivalent functions to allow users to easily perform module detection, and then perform functional enrichment analysis on these modules.

The future plans for NetworkAnalyst involve two directions - to increase its support for more organisms and to add more features to its visualization. In particular, we will gradually add support for PPI network analysis of other model organisms. For non-model organisms, we will allow users to upload their custom-defined networks together with the expression data. For visualization features, we are currently adding a feature to visualize correlations between co-expressed genes as different edge weights (thickness). We also intend to add a new feature to allow users to manually rearrange node positions when networks are small (e.g. the final extracted modules). In addition, we also plan to collaborate with other groups to implement a network layout method based on subcellular localization annotation as implemented in cerebral (34).

## COMPARISON WITH OTHER EXISTING TOOLS

Several web-based tools have been developed in the past few years for network analysis and visualization. Some of them are specialized for comparing and analyzing networks themselves such as GraphWeb (35), NeAT (36), etc. Here, we choose to compare only with those tools that are designed to analyze data from gene expression experiments within the context of biological networks. The detailed comparisons are listed in Table 1. Based on the comparisons, it is clear that NetworkAnalyst provides better support in terms of differential gene expression data analysis, as well as network construction. It is also currently the only web-based application that supports online interactive visualization of large biological networks without the need to install any plugins.

**Table 1.** Comparison with other web-based network analysis tools

Tools	NetworkAnalyst	InnateDB	STRING	R Spider	VisANT
Implementation	Web	Web	Web	Web	Java Applet
Supported organisms	Human and mouse	Human, mouse and cow	>1100 organisms	Six model organisms	112 species
**Data processing**					
Input	Gene or protein list, single or multiple expression data	Gene or protein list	Protein list	Gene or protein list	Gene or protein list
Probe annotation	Affymetrix, Illumina, Agilent	No	No	Affymetrix	No
Differential analysis	Yes	No	No	No	No
**Network construction**					
Interaction type	PPI	PPI	PPI	Metabolic, signaling	PPI, metabolic, drug, disease
Interaction distance	Zero, first or higher order	Zero or first order	Zero, first or higher order	Zero, first or second order	Zero, first or higher order
**Network analysis and visualization**					
Interactive visualization	Yes	Delegated to Cytoscape	Built-in visualization tools are suitable for small networks; Cytoscape plugins are available for visualizing large networks		Yes
Topology analysis	Yes				Yes
Module analysis	Yes				Yes
Functional analysis	Yes				Yes
Result download	Edge list, PNG, PDF, GraphML				Edge list, VisML, SVG

The URL for each tool is given below:

NetworkAnalyst: http://www.networkanalyst.ca.

InnateDB: http://www.innatedb.com.

STRING: http://string-db.org.

R Spider: http://mips.helmholtz-muenchen.de/proj/rspider.

VisANT: http://visant.bu.edu/.

## CONCLUSIONS

Biological network analysis is a powerful approach to gain insights into complex diseases or biological systems. Even in diseases primarily driven by a single gene mutation such as cystic fibrosis, network-based approaches can often reveal novel pathogenesis pathways and identify potential therapeutic targets (37). Although there has been significant progress in the development of web-based tools that support processing and statistical analysis for data from various omics experiments (38,39), user-friendly and web-based tools to assist bench researchers and clinicians in performing complex network analysis are still lacking. In addition, integrating multiple gene expression datasets (meta-analysis) have become increasingly applied for improved biomarker identification and biological understanding (40,41). In this paper, we introduced NetworkAnalyst, an easy-to-use web-based tool designed to assist bench researchers and clinicians to perform various common tasks involved in PPI network analysis starting from gene expression data. Its key features include integration of data processing for various gene expression data analysis, flexible network construction and interactive network visualization. Particular attention has been paid to reduce the most criticized ‘hairball’ effect when viewing a large network. For instance, users can control the starting network size using any of the four procedures during network construction; during network visualization, users can hover the mouse over any node to view only the node and its direct neighbors. Finally, users can select important hubs or modules, and extract them from the parent network. At any time, users can perform *in situ* functional analysis for nodes highlighted in a network. By integration of gene expression, protein interaction and functional annotation, we hope to help researchers to decipher complex diseases and arrive at novel biological insights.
